# Editorial: The psychology of hope

**DOI:** 10.3389/fpsyg.2025.1671248

**Published:** 2025-12-17

**Authors:** Tharina Guse, Andreas Krafft, Angelina Wilson Fadiji

**Affiliations:** 1 Department of Psychology, University of Pretoria, Pretoria, South Africa; 2 Institute for Systemic Management and Public Governance, University of St Gallen, St. Gallen, Switzerland; 3 Department of Psychology, School of Applied Social Sciences, De Montfort University, Leicester, United Kingdom

**Keywords:** hope, hope in context, hope interventions, hope research foci, transdisciplinary hope, transformative research, hope as emotion, collective hope

Since the inception of the field of positive psychology, with its interest in understanding factors that make people thrive, hope has featured prominently. Hope is essentially about thoughts, emotions and behaviors related to the future. How individuals view the future, will also influence how they act in the present (Pleeging et al., 2022). Hope thus offers a mechanism for navigating adversity and envisioning possibility, making it an important construct in furthering knowledge and developing interventions to promote wellbeing. However, as the field of positive psychology matures into its third wave and beyond (Lomas et al., 2021; Wissing, 2022) there are calls to revisit our understanding of key wellbeing concepts. More specifically, (van Zyl and Dik 2025) proposed a paradigm shift in examining positive psychology constructs and processes, arguing for a move toward person-centered, ideographic, bottom-up approaches that are more inclusive and context-sensitive. It may be an opportune time to consider hope research against this backdrop.

Snyder's (2000); (Snyder 2002) cognitive-motivational hope theory made a seminal contribution to understanding individual thought processes that sustain motivation in reaching goals. It remains one of the most influential psychological theories on hope (Gallagher, 2023). While there were early calls for alternative conceptualizations of hope (e.g., Averill and Sundararajan, 2005; Webb, 2007), and frameworks outside of psychology were developed (e.g., Herth, 1991, 1992), more nuanced models on the nature and measurement of hope only started to emerge over the past decade (e.g., Krafft et al., 2019, 2023b; Scioli et al., 2016). There is also increased interest in broader, transdisciplinary understandings of hope (e.g., Colla et al., 2022; Krafft et al., 2023b; Pleeging et al., 2022). Further, there have been proposals to examine hope beyond individualistic, Western contexts (Bernardo, 2010; Bernardo and Ramos; Krafft et al., 2023b; Thomas et al., 2023) and to broaden our understanding of the sources of hope.

This volume of *The Psychology of Hope* emanated from our interest in further unraveling how hope can be understood in different contexts and through different conceptualizations. Our aim was to expand theoretical knowledge of hope, traversing from existing individualistic, cognitive-motivational perspectives to broader systems, cultural contexts, communities, and the environment. Moreover, we aimed to elicit knowledge on strategies to strengthen and sustain hope in different contexts in a manner that challenges traditional individual assumptions and advocates for integrating social, environmental, and communal dimensions. In this editorial, we draw upon the contributions to the volume to propose avenues toward expanding hope research. We also consider recent frameworks of hope as a group-based emotion (Cohen-Chen and Pliskin, 2025), cross-cultural findings on hope (Krafft et al., 2023b) as well as van Zyl and Dik's (2025) proposed person-centered, ideographic approach in considering future research. We argue that there is a need to revisit the foci (threads) and methods (looms) in weaving the tapestry of understanding hope. While examining hope on an individual level remains important, exploring the experience and expression of hope in relational, group and collective contexts require attention. We also highlight methodological and theoretical considerations for future research. Finally, we propose a transdisciplinary umbrella of *hope studies*, to allow for a more comprehensive understanding of how beliefs and feelings about the future support wellbeing for all. As we consider future hope research, two questions guide this editorial: Who and what should we examine when we study hope? How can our methods more comprehensively capture the complexity of hope? Whilst not exhaustive, we attempt to provide some answers.

## Who, and what, should we examine in hope research?

### Living hope: individual experiences of hope and hoping

The past two decades of hope research has generated substantial insights on individual levels of hope as well as correlations between hope and important psychological outcomes. For example, higher levels of hope in adolescence are associated with higher levels of psychological wellbeing in adulthood (Long et al., 2024) and serve as a protective factor on times of crisis, such as the COVID-19 pandemic (Krafft et al., 2023a). However, these findings mostly emanated from existing top-down approaches to studying hope, relying on survey research, averages and universal models of hope (see van Zyl and Dik, 2025). While some scholars have explored experiences of hope (e.g., de Pretto et al., 2020; Møller et al., 2024) and lay definitions of hope (Feldman et al., 2023) there is a need for more in-depth, ideographic studies on the subjective experience of hope, the process of hoping and the meaning of hope. For example, when do individuals experience hope and how would they describe it? What does the process of hope entail? Moreover, what enables people to hope, and what dampens hope? This might encompass looking more closely at collectivist and indigenous communities and exploring how communal and spiritual dimensions of hope manifests in these contexts (see Thomas et al., 2023). There is ample opportunity to explore the rich, lived experiences of individuals in weaving the tapestry of hope.

### Hoping together: relational, group, and collective hope

Hope is not only an individualized experience but also materializes in relational contexts. It is important to consider the interconnectedness of the individual, family, community, and environment in maintaining a hopeful outlook toward the future (Bryce and Fraser, 2023; Møller et al., 2024; Wissing, 2014). (Colla et al. 2022) argued that we need to move beyond the limitations of the individualistic and reductionistic investigation of hope by situating it as a systemic phenomenon that acknowledges its contextual networks, complexity, and possibly emergent nature. Similarly, (Pleeging et al. 2022) argued that social connections and institutional, political, cultural and economic contexts could influence hope. However, how people understand and experience hope, particularly in relation to interpersonal connections and broad contexts, still needs to be further examined. Expanding on van Zyl and Dik's (2025) proposed ideographic approach, researchers could explore hope in relational terms, taking into consideration the lived experiences of individuals in different relational contexts.

Such research could focus on three levels: First, we could examine how relational and group contexts influence individual levels of hope, hope processes and hope outcomes. For example, (Bernardo 2010, 2015) postulated that external agents such as social groups and spiritual dimensions may influence the way individuals think about goals and pathways to goals. While Bernardo and Ramos (this volume) advocate for integrating cultural dimensions into cognitive theories of hope, these processes still need to be further investigated. To explore these relational dimensions would require measurement tools that accurately capture the multi-layered complexity of assessing how different agents contribute to a specific individual's experience of hope. Not only do we need to capture the role of these different agents, but we might need to interrogate how each of them differentially contributes to or possibly even diminish hope.

Second, it is important to examine how hope is expressed and experienced in group settings. Research on hope from a social-psychological perspective showed that hope can function as a group-based emotion in the context of intra- and intergroup relations. Specifically, hope can lead to the attainment of both positive and negative outcomes (Cohen-Chen and Pliskin, 2025). For example, the authors found that hope is functional for promoting positive intergroup attitudes but may be less optimal for inspiring collective action to oppose social inequality. In this volume, Leshem and Halperin provide a framework for understanding the emotional drivers, deep-seated wishes or hopes that may sustain groups and activists who are engaged in long-term social or political conflicts. This line of research mostly emanated from contexts of intractable conflicts. Therefore, more studies are needed to understand group-based hope in other cultures and contexts such as marginalized groups and non-Western contexts. Another imminent research gap is to explore what hope looks like among people faced with systemic disadvantages and oppression, as well as disabled or incarcerated individuals. It is necessary to understand how group-based hope would manifest in the face of adversities that are embedded in the way the society is organized. For example, can hope support and foster actions targeted at navigating structural barriers?

Finally, the notion of collective hope (Krafft; Krafft et al., 2023b) incorporates broader social domains and culturally shared worldviews and their relationship to hope. In this volume, Krafft argues that hope encompasses more than individualistic goal-oriented dimensions and incorporates broader social domains and culturally shared worldviews. He supports his view by presenting findings on hope and worldviews among Italian and French-speaking samples in Switzerland. However, we still need to understand what collective hope entails from individuals' and groups' perspectives, and how it is expressed and sustained. In sum, how hope materializes in groups and communities, and how hope can be mobilized in various contexts and through various relationships, needs to be further explored. Like research has progressed in measuring societal happiness, similar approaches could be implemented to measuring and promoting hope.

### Feeling hopeful: the affective dimensions of hope

While most studies on hope have focused on its cognitive dimensions, several authors proposed that hope is primarily an emotion (e.g., Averill et al., 1990; Bruininks and Malle, 2005) or that hope has an emotional component that could influence goal-directed behavior (Feldman and Jazaieri, 2024). Yet, there is still a gap in research that explains the mechanisms and dynamics of hope as an emotion. (Fredrickson 1998, 2013) identified hope as one of ten most experienced positive emotions and proposed that hope contributes to building psychological resources through encouraging individuals to aspire to a better future. However, empirical studies on hope in the context of broaden and build theory (Fredrickson, 1998) are still limited. For example, can hope be induced in the moment through specific activities, and what are the behavioral outcomes? How, and when does hope broaden action-thought repertoires? Would it be possible to explore this from a neuroscience perspective, given existing neurological evidence showing that specific parts of the brain are activated when positive emotions are being experienced (Alexander et al., 2021)? More recently, (Edwards et al. 2024, 2025) initiated research on inducing hopeful feelings and its associated outcomes, but more research is required regarding the cultural applicability of inducing hope in different contexts. This is quite important as (Appiah 2022) highlighted the unintended harm that wellbeing interventions could have when they are not properly tailored for a specific context.

(Cohen-Chen and Pliskin 2025) proposed a nuanced perspective of hope as an emotion, specifically in group-based contexts. The authors examined hope through a valence and function framework suggesting that hope can be categorized based on two orthogonal dimensions: the pleasantness of the emotional experience (valence) and the social outcomes (function). This framework goes beyond existing assumptions that hope is positive and highlights how group-based hope may lead to negative outcomes and emotions. In the individual domain, less is known about the valence and function of hope. If we consider that hope is also an ambivalent emotion (Lomas, 2017), and can co-occur with negative affect (Gasper et al., 2020), more research is required regarding the experiences and outcomes of hope as an emotion. This must also be adequately captured in measurement approaches.

### Talking about hope: hope discourses

While we have learned much about the individual mechanisms and correlates of hope, it can be fruitful to examine how hope is created in language and conversations. Some scholars proposed that hope is a socially constructed phenomenon (e.g., Åhlvik et al., 2024). What can we learn about the phenomenon of hope through analyzing messages of hope and discourses of hope? How does this link to collective hope and action toward a better future? How is hope constructed in social situations? For example, messages that evoke hopeful future attitudes seem to promote pro-environmental behavior (Schneider et al., 2021). Hope discourse therefore has social implications (Åhlvik et al., 2024), and there is a need to examine more closely how hope is created or constructed in naturally occurring conversations. Further, it is important to explore the outcomes of hope discourses in the context of socio-political issues such as climate change and geo-political conflict.

### Hope in context

Hope is anchored in beliefs and feelings about a desired future. However, these beliefs and affective experiences may be shaped by culture and context. It is therefore important to place context as a key element in examining hope. Recent survey research demonstrated differences in levels of hope among participants from different cultures and contexts (e.g., Krafft et al., 2023b; Slezácková et al., 2021). Qualitative enquiry among South African adults living in adversity indicated that context is paramount when studying hope beyond value-neutral, self-focused Western contexts. Specifically, in contexts where external societal support is lacking and inequality persists, individuals anchor their hope in relational and transcendent sources (Thomas et al., 2023). In this volume, Mason demonstrates how hope is interwoven with social responsibilities and collective aspirations in a collectivist context. Similarly, Bernardo and Ramos point out that empirical research on hope often yielded divergent findings in diverse cultural contexts, particularly regarding the two-factor structure of hope. The authors expand on Bernardo's (2010) earlier work on “locus-of-hope,” differentiating between internal (disjoint) and external (conjoint) sources of hope, which are crucial in collectivist cultures where spiritual beliefs, religious practices, and family-relational processes are significant sources of hope. These dimensions could be fruitfully explored by engaging with individuals, groups and communities.

The importance of context is also seen in the nature of goals and hopeful thinking patterns that individuals develop. (Khumalo et al. 2022) argued that prioritization of tertiary education and employment among South African university students was shaped by the need to secure a better material future and support their families, capturing the kinds of obstacles these young people needed to navigate. Also in this volume, Chan et al. provides evidence that hope, viewed as a cognitive-motivational construct, served as a protective factor for sleep quality among university students in Hong Kong, while the culturally relevant construct of negotiable fate did not relate to sleep. This highlights the complex interplay between context and specific conceptualizations of hope. While research on hope in context has emerged substantially over the past few years, it is evident that more research is needed to explicitly consider how context influences hope, as hope may be situated in the particular place people find themselves to be at a particular time (Counted and Newheiser, 2024) as well as the broader societal context (Krafft et al., 2023b; Thomas et al., 2023).

### Hope outcomes

A large body of research has reported on the positive outcomes associated with hope, including higher levels of subjective wellbeing (e.g., Pleeging et al., 2021), positive health outcomes and health behaviours (e.g., Abeyta, 2023; Scioli et al., 2016) and lower levels of distress (e.g., Slezackova et al., 2023). An explanation for the social, psychological and biological mechanisms underlying these outcomes need to be explicated in broader contexts. Further, hope has substantive prosocial outcomes (Schornick et al., 2023; Thomas et al., 2023). However, less is known about the negative outcomes of hope. (Cohen-Chen and Pliskin 2025) discussed how hope as a group-based emotion can lead to dysfunctional social outcomes (“doing bad”) and unpleasant individual experiences (“feeling bad”). More specifically, they argued that hope could act as a blindfold, leading to risk-taking and enabling acceptance of systemic injustice, or to psychological distress when induced in contexts of high uncertainty. Future research could explore negative outcomes associated with different conceptualizations of hope in various contexts and among various individuals, groups and communities.

### Fostering hope: hope interventions

Given the multiplicity of hope theories and conceptualizations, it is important to critically examine the development and applications of hope-based strategies and interventions. Drawing mainly on cognitive conceptualizations of hope, findings suggest that hope is malleable (Belfer et al., 2025). Strategies to enhance hope have been implemented in several contexts, including health care (e.g., Feldman and Corn, 2023), psychotherapy (e.g., Joubert and Guse, 2022) and education (e.g., Alam and Mohanty, 2024). In this volume, Scioli et al. present an interdisciplinary, hope-centred intervention for adolescents, designed around the fundamental needs for attachment, survival, mastery, and spirituality. Findings showed significant increases in hope, self-acceptance, and social coping, along with a reduction in depression. Further, the study demonstrates that hope interventions may be efficient, even when delivered by paraprofessionals. Also in this volume, Zhu et al. investigate how perceived peer support, academic hope, and professional identity jointly facilitate college students' academic adjustment. They provide empirical evidence for developing targeted support strategies that aim to enhance academic performance and psychological wellbeing. The findings highlight academic hope as the most significant mediating factor in promoting academic adjustment.

However, the focus of hope interventions has mostly been on individual hope and outcomes. More research is required on ways of instilling, enhancing and sustaining hope in groups and communities. It is also important to understand drivers and sources of hope in designing interventions to foster and sustain hope in different contexts. In this volume, Laranjeira et al. propose the concept of ecological hope as a dynamic, multifaceted, cognitive-emotional-motivational state that contributes to understanding hope in the context of large-scale challenges, such as climate change. They further present practical steps for promoting ecological hope in education for sustainable development. Also in this volume, Slezackova et al. report on the crucial role of hope in the Russia-Ukraine war. Specifically, hope seems to act as a catalyst in transforming negative experiences into growth opportunities. Therefore, hope has potential as a target for psychological interventions to promote resilience and wellbeing in the face of adversity. However, the nature and outcomes of such interventions still need to be explored.

Finally, research needs to explore whether there may be instances where implementing hope interventions may be contra-indicated. Hope involves the desire for an outcome that may not be achieved and could potentially be accompanied by unpleasant affect, such as fear. In such instances, interventions to enhance hope could also increase fear (Gasper et al., 2020). It is therefore important to examine specific contexts and populations where unintentional exacerbation of fear while enhancing hope could potentially have an iatrogenic effect. Similarly, the applicability of hope interventions in non-Western contexts need to be further examined, as these may not always be suitable when the focus is on individualistic conceptualizations of hope (Appiah, 2022).

## How could we examine hope?

### Implementing contextually relevant approaches

Hope research may suffer similar limitations as wellbeing-research, which tended to be prescriptive, individualistic and expert-led, with less attention given to contextual, deeper and richer personal understandings of wellbeing (van Zyl and Dik, 2025). We suggest that scholars could expand existing approaches to studying hope and move beyond averages, such as levels and correlates of hope, to explore nuanced experiences, expressions and practices of hope. In this volume, Mason contributes by examining hope among students within a collectivist context using a qualitative approach. (Thomas et al. 2023) made an important contribution in examining lived experiences of hope among adults living in South African township, but there is still a need for further studies utilizing similar research approaches in other contexts.

Further, it may be timely to adopt an explicit relational lens in studying hope. From a relational perspective (White and Jha, 2023), social contexts, connections and interactions in everyday life influence experiences of wellbeing. The same may be true for experiences of hope. Approaches could implement relational thinking and consider how the complexities of individual and collective perspectives inform the inter-relations and connections among individuals and, eventually, their experiences of hope (or lack thereof). Participatory action research (Keahey, 2021) and creative research approaches (Kara, 2023) could be useful to understand the complexity of relational aspects in experiencing hope.

Finally, there is scope for examining hopeful cognitions, emotions and experiences from a neuroscience perspective. Research focused on the neural correlates of positive emotions, including hope, is still considered nascent compared to the research on negative emotions (see Alexander et al., 2021) while neuroimaging studies on hope as cognitive process are only starting to emerge (Dasgupta et al., 2023).

### Adopting methodological pluralism and engaging in transformative research

Hope research could benefit from adopting methodological pluralism, incorporating more diverse methodologies. Such an approach could combine quantification of hope, explore lived experiences qualitatively and model complexity using network analysis while also reflecting on its philosophical aspects. This will allow for richer insights, greater inclusivity, improved validity and innovation in examining hope. For example, (Thomas et al. 2023) implemented qualitative research using both top-down and bottom-up analyses to explore hope among individuals living in a resource-constrained African context.

Future studies could also explore the applicability of Krafft et al.'s (2019; 2023b) multidimensional model and Scioli et al.'s (2011) multidimensional network model of hope in similar contexts. Building on recent calls to engage in transdisciplinary research (Krafft et al., 2023b; Pleeging et al., 2022) more focused studies combining expertise from philosophy, religion and psychology, among others, is needed to capture the complexity linked to the science of hope.

Overall, we view the need to engage in transformative research in understanding and promoting hope as crucial to the development of the field. Transformative research aims to combine knowledge production with co-creating intentional change toward a sustainable trajectory (Horcea-Milcu et al., 2024). It further addresses societal problems through developing social and scientific knowledge which could support sustainability transitions (Wittmayer et al., 2021). Applied to hope research, a transformative turn would imply a shift from descriptive analysis to active, change-making inquiry. For example, research could examine hope as a leverage point and identify where and how hope could accelerate change toward a better future, rather than observing its general presence. Another possible research approach would be to engage stakeholders such as communities, NGOs, and policymakers in co-designing studies. This will enable researchers to understand what type of hope is needed and functional in addressing specific crises or challenges, for example, climate change or inequality (see Horcea-Milcu et al., 2024).

### Utilizing advances in data science

Recently, wellbeing research has expanded beyond traditional quantitative and qualitative approaches to examine various constructs at scale using advanced computational methods. For example, during the COVID-19 pandemic, (Sarracino et al. 2023) captured national affective mood in 10 countries using publicly available, high-frequency text data (Twitter) instead of traditional, time-consuming surveys. The researchers treated a vast number of social media posts as a continuous stream of data which is provided almost instantaneously. This enabled them to track wellbeing dynamics daily. Natural Language Processing (NLP) (see Mishra et al., 2025), a form of machine learning, was used to conduct sentiment analysis, which involves automatically interpreting feelings and attitudes in the text. This approach allows researchers to capture momentary wellbeing or affective states. (Greyling and Rossouw 2025) similarly proposed an innovative methodological approach using Google Trends™ to develop a real-time happiness index. This involved extracting emotion words from information search queries and using machine learning algorithms to analyse their correlation with happiness on a population level.

Following a similar approach, hope research could include sentiment analysis of online forums and social media, using NLPs to explore feelings and cognitions about the future. Large-scale datasets offer the possibility to investigate cognitive and affective dimensions of hope qualitatively using generative AI analyses (Choudhury et al., 2025), providing rich ideographic data (van Zyl and Dik, 2025) as well as population-level indices of hope. Further, large-scale data could enable research on messages of hope or hope communication (see Åhlvik et al., 2024). Following Greyling's and Rossouw (2025) approach to measure happiness in real time through analysing information seeking queries, it is plausible to develop a real-time hope barometer to obtain insight into national levels of hope. Moreover, a Big Data approach offers sophisticated tools for monitoring hope, by providing continuous information on the collective hope of large populations. It can be especially valuable to investigate state hope in specific contexts and timeframes, such as socio-political instability, health crises and economic downturns (see also Rossouw and Greyling, 2022, 2025). In using Big Data, we might also consider the role of social media in promoting or diminishing hope during widespread adversity, for instance, a pandemic or even the use of digital media in implementing community-wide digital intervention to support mental health promotion.

## Conclusion

The contributions in this volume offer an exploration of hope's complex and interwoven nature. While the authors examine hope through its established components, they also introduce interdisciplinary, transdisciplinary, and relational aspects. The findings of the studies in this issue further reinforce the complexity associated with understanding positive constructs and their diverse range of applications across various personal and social domains. Hope is not a single thread, but a tapestry of cognitions, emotions, relationships, culture, and context. However, existing research has mostly focused on the cognitive, individualistic strands, adopting top-down research approaches and weaved limited understandings of what hope entails. It is now time to weave a more expansive and inclusive tapestry of hope scholarship.

In widening perspectives on studying hope, there is also the opportunity for broadening toward a transdisciplinary field of *hope studies* that considers the complexity of how individuals think about, feel, and strive toward better futures. In keeping with the metaphor of weaving, we need to consider which threads of hope (e.g., individual, relational, collective, contextual) must be more fully explored, and how we can implement methodological approaches as the weaving apparatus to better capture the nature of hope. In doing so, the field could advance to a richer, more inclusive reflection of diverse experiences of hope and contribute to efforts to support wellbeing across contexts.
